# Outcomes of Mobile Health Use in Sinonasal Surgery: Retrospective Cohort Study

**DOI:** 10.2196/75403

**Published:** 2026-07-21

**Authors:** Heli Majeethia, Akshay R Prabhakar, Justina R Varghese, Najm S Khan, Roshan Dongre, Vincent Provasek, Faizaan I Khan, Zain Mehdi, Omar G Ahmed, Masayoshi Takashima

**Affiliations:** 1 Department of Otolaryngology - Head & Neck Surgery, Houston Methodist Academic Institute, 6550 Fannin Street, Houston, TX, United States, 1 (346) 238-1633; 2 Rutgers New Jersey Medical School, Rutgers, The State University of New Jersey, New Brunswick, NJ, United States; 3 School of Engineering Medicine, Texas A&M University, Houston, TX, United States; 4 Texas A&M College of Medicine, Texas A&M University, Houston, TX, United States

**Keywords:** mobile health, telehealth, patient-centered care, patient satisfaction, patient empowerment, patient engagement, patient involvement, hospital stay, communication programs

## Abstract

**Background:**

Mobile health (mHealth) technologies are increasingly integrated into perioperative care to enhance patient engagement and communication. Prior studies in surgical and medical specialties suggest that mHealth platforms may be associated with reductions in hospital stay and readmissions; however, evidence supporting their impact in otolaryngology, particularly in sinonasal surgery, remains limited.

**Objective:**

The aim of this study was to evaluate the association between perioperative enrollment in CareSense, a patient-facing mHealth platform, and postoperative health care utilization outcomes, including hospital readmissions, emergency department (ED) visits, and length of stay (LOS), among adults undergoing sinonasal surgery.

**Methods:**

This is a retrospective cohort study performed at a single tertiary care academic medical center between May 2021 and January 2024. All adult patients (≥18 years) who underwent sinonasal surgery with two fellowship-trained rhinologists during the study period were included. CareSense was offered to all patients at the time of surgical scheduling, and enrollment was voluntary. Patients were categorized into CareSense participants and nonparticipants. Primary outcomes were all-cause hospital readmissions and ED visits within 30, 60, and 90 days following surgery. Secondary outcomes included the length of hospital stay among readmitted patients. Clinical, demographic, and outcome data were obtained through retrospective electronic health record review. Univariate analyses compared outcomes between groups, and multivariable logistic regression using generalized estimating equations was performed to estimate the association between CareSense participation and outcomes while adjusting for age, sex, hypertension, and diabetes.

**Results:**

A total of 1135 patients were included, of whom 340 (30%) enrolled in CareSense and 795 (70%) did not. Compared with nonparticipants, CareSense participants had lower adjusted odds ratio (OR) for readmission for any cause at 30 days (OR 0.24, 95% CI 0.08‐0.75; *P*=.007), 60 days (OR 0.40, 95% CI 0.19‐0.83; *P*=.01), and 90 days (OR 0.54, 95% CI 0.29‐0.99; *P*=.04). Among patients who were readmitted, mean LOS was shorter in the CareSense group than in the nonparticipating group (0.17 vs 1.68 d; *P*<.001). The majority of readmissions in both cohorts were unrelated to complications of the index sinonasal procedure.

**Conclusions:**

This study demonstrates the benefit of CareSense in lowering postoperative readmission rates and LOS for sinonasal surgery patients, illustrating the role of medical health technology in improving patient care and quality outcomes. Perioperative enrollment in a patient-facing mHealth platform was associated with lower postoperative health care utilization and shorter hospital length of stay following sinonasal surgery. Given the voluntary nature of enrollment and the observational design, these findings should be interpreted as observation findings and hypothesis-generating for prospective studies to more definitively assess the causal impact of mHealth interventions and to identify which components of digital perioperative care most effectively improve outcomes in otolaryngologic surgery.

## Introduction

Continuous improvement in patient care and clinical outcomes remains a central goal in health care, and advances in digital technology have created new opportunities to support this aim by improving communication, education, and patient engagement. The World Health Organization defines mobile health (mHealth) as “medical and public health practice supported by mobile devices, such as mobile phones, patient monitoring devices, personal digital assistance, and other wireless devices” [1,2]. As mobile phone usage steadily increases worldwide, leveraging this development offers the potential for rapid dissemination of health information and remote patient engagement, which may enhance medical care as evidenced during the COVID-19 pandemic [3].

Over the past decade, mHealth interventions have been increasingly incorporated into routine care across multiple specialties [4]. Emerging evidence suggests that these platforms may be associated with improvements in health care utilization outcomes, including reduced length of stay and lower readmission rates in orthopedic and cardiovascular populations [5-8]. In otolaryngology, mHealth tools have been adopted for perioperative communication and symptom monitoring; however, evidence evaluating their association with postoperative health care utilization remains limited, particularly for patients undergoing sinonasal surgery [9-11].

CareSense is a patient-facing mHealth platform designed to support perioperative care through automated educational content and reminders delivered via text message or email, with parallel clinician-facing dashboards for monitoring patient-reported information. The platform aims to improve patient preparedness and adherence with visits and surgeries by facilitating perioperative education and communication, while enabling tracking of patient progress and satisfaction. The aim of this study was to evaluate the association between perioperative enrollment in CareSense and postoperative health care utilization outcomes, including hospital readmissions, emergency department visits, and length of hospital stay, among adults undergoing sinonasal surgery.

## Methods

### Study Population

A retrospective observational cohort study was conducted at a single tertiary academic medical center between May 2021 and January 2024. Beginning May 2021, the Department of Otolaryngology–Head and Neck Surgery at Houston Methodist Hospital began offering CareSense (MedTrak, Inc) to all patients undergoing sinonasal surgery. Clinical, demographic, and outcome data were obtained through retrospective review of the electronic health record (EHR). All patients ≥18 years of age who underwent sinonasal surgery with two fellowship-trained rhinologists were included. Patients were divided into 2 groups: those who elected to participate in CareSense and those who did not. Enrollment in CareSense was voluntary and occurred at the time of surgical scheduling.

### Ethical Considerations

This study was reviewed and approved by the Houston Methodist Research Institute Institutional Review Board (PRO00036908) with a waiver of informed consent due to its retrospective design and use of existing clinical data. All data were analyzed in a deidentified format to protect patient privacy and confidentiality. No compensation was provided to the participants.

### Mobile Health Technology

CareSense is a digital platform used to deliver standardized perioperative educational content and automated reminders to patients undergoing surgery. Enrolled patients received procedure-specific “pathways,” defined as preset configurations of communication and information delivery tailored to the planned surgery (eg, sinus surgery or septoplasty). Each pathway provided surgery-specific perioperative instructions and educational materials, including preoperative preparation, postoperative expectations, warning signs of complications, and medication courses. Message timing and content were predefined and delivered consistently across enrolled patients via text message or email. Communication formats included brief text notifications, longer-form educational emails, and physician-defined survey questions, with selected responses generating alerts within a clinician-facing Care Navigator dashboard. Details of the sinus surgery and septoplasty pathways are provided in MultimediaAppendices 1  2, respectively. Patients without access to a mobile phone or email address were not eligible for enrollment.

### Outcome Measures

The primary outcomes evaluated were readmission rates and emergency visits to any of our hospital system sites within 30, 60, and 90 days of discharge following sinonasal surgery. Readmissions were defined as any unplanned inpatient or observation admission occurring after surgery. Emergency department (ED) visits were defined as encounters that originated in the ED and resulted in discharge without inpatient admission. Outcome ascertainment was performed through system-wide EHR review, capturing events occurring within the same integrated health system. The secondary outcome was postoperative length of stay (LOS) among patients who were readmitted, used as a surrogate for the complexity and acuity of the readmission. Outcomes were assessed at fixed postoperative intervals; variation in individual follow-up duration beyond these windows was not evaluated. All patients were followed through the EHR for a minimum of 90 days postoperatively within the integrated health system, with outcome ascertainment limited to encounters captured during these predefined intervals.

### Statistical Analysis

Demographic and comorbidity data were available for all patients. Differences in baseline patient characteristics between CareSense participants and nonparticipants were assessed using an independent-samples *t*-test for continuous variables and *χ*^2^ tests for categorical variables. Univariate analyses using *χ*^2^ testing were performed to compare outcome measures between the two cohorts. Given the low frequency of procedure-related events, analyses of surgery-related readmissions were conducted descriptively rather than through inferential subgroup modeling. To partially address confounding related to voluntary enrollment, multivariable logistic regression using generalized estimated equations (GEE) was used to estimate the association between CareSense participation and postoperative outcomes while adjusting for prespecified covariates, including age, sex, hypertension, and diabetes. Covariates were prespecified a priori based on clinical relevance and prior literature evaluating postoperative health care utilization [12-14]. Odds ratios (ORs) with 95% CIs were reported. All statistical tests were two-sided, with an α level of .05 considered statistically significant. Univariate analyses were performed using Jamovi [15], and multivariable analyses were conducted in RStudio (version 4.3; R Core Team, 2023).

## Results

### Study Population

A total of 1135 patients underwent sinonasal surgery from May 2021 to January 2024. Among these patients, 340 (30%) elected to participate in CareSense, whereas 795 (70%) did not participate. Enrollment in CareSense increased over time, with an 80.7% increase from 2021 to 2024.

Of the 340 patients enrolled in CareSense, 202 (59%) were assigned to the sinus surgery pathway and 138 (41%) to the septoplasty with turbinate reduction pathway (Multimedia Appendix 1 and Multimedia Appendix 2, respectively). Patient demographics and baseline characteristics are summarized in Table 1.

**Table 1.** Sample patient characteristics of nonparticipating and participating patient groups.

Variables	Patients, n (%)		*P* value
	Nonparticipating (n=795)	Participating (n=340)	
Mean age in years (SD)	51.1 (17.8)	47.1 (17.2)	<.001
Sex			.04
Female	375 (47.2)	138 (40.6)	
Male	420 (52.8)	202 (59.4)	
Ethnicity			
Declined	14 (1.8)	14 (4.1)	.02
Hispanic or Latino	133 (16.7)	49 (14.4)	.33
Not Hispanic or Latino	648 (81.5)	277 (81.5)	.99
Race			
Asian	73 (9.2)	41 (12)	.14
Black	109 (13.7)	37 (10.9)	.19
White	555 (69.8)	237 (69.7)	.97
Declined	48 (6)	20 (5.9)	.92
Other	10 (1.3)	5 (1.5)	—^a^
Comorbidity			
Asthma	176 (22.1)	78 (22.9)	.77
Atherosclerosis	127 (16.0)	42 (12.4)	.12
COPD^b^	64 (8.1)	26 (7.6)	.82
Diabetes type 2	150 (18.9)	41 (12.1)	.005
GERD^c^	286 (36.0)	115 (33.8)	.49
Heart failure	52 (6.5)	16 (4.7)	.23
Hypertension	365 (45.9)	127 (37.4)	.008
Hyperlipidemia	339 (42.6)	135 (39.7)	.36
Illicit drug use			
Yes	27 (3.4)	11 (3.2)	.89
No	710 (89.3)	314 (92.4)	.11
Alcohol use			
Yes	395 (49.7)	187 (55.0)	.10
No	364 (45.8)	148 (43.5)	.48
Tobacco use			
Yes	49 (6.2)	20 (5.9)	.86
Quit	168 (21.1)	79 (23.2)	.43
Never	547 (68.8)	238 (70.0)	.69
Smoking or tobacco use			
Current	49 (6.2)	21 (6.2)	.86
Former	165 (20.8)	77 (22.6)	.48
Never	550 (69.2)	240 (70.6)	.64

a Not available.

b COPD: chronic obstructive pulmonary disease.

c GERD: gastroesophageal reflux disease.

Overall, the mean age of participating patients was 47.1 (SD 17.2) years, and that of nonparticipating patients was 51.1 (SD 17.8) years (*P*<.001). Males accounted for 420 of 795 (52.8%) patients in the nonparticipating group and 202 of 340 (59.4%) patients in the participating group (*P*=.04). There were no statistical differences in race and ethnicity between groups.

The nonparticipating group had a higher prevalence of hypertension (365/795, 45.9% vs 127/340, 37.4%, *P*=.008) and type 2 diabetes (150/795, 18.9% vs 41/340, 12.0%, *P*=.005). There were no significant differences between groups with respect to asthma, atherosclerosis, chronic obstructive pulmonary disease, gastroesophageal reflux disease, heart failure, and hyperlipidemia. Self-reported alcohol use, tobacco use, and illicit drug use did not significantly differ between groups.

### Outcome Measures

Readmission rates are presented in Table 2. At 30 days postoperatively, readmission rates were 5.8% (46/795) in the nonparticipating group and 1.2% (4/340) in the participating group (*P*<.001). At 60 days, readmission rates were 7.9% (63/795) in the nonparticipating group and 2.9% (10/340) in the participating group (*P*=.002). At 90 days, readmission rates were 8.9% (71/795) in the nonparticipating group and 4.1% (14/340) in the participating group (*P*=.005).

**Table 2.** Comparing the likelihood of readmissions and emergency visits between patients who were and were not enrolled in CareSense.

Outcome variables	Patients, n (%)		*P* value
	Control (n=795)	Intervention (n=340)	
Readmission (days)			
30	46 (5.8)	4 (1.2)	<.001
60	63 (7.9)	10 (2.9)	.002
90	71 (8.9)	14 (4.1)	.005
Emergency visit (days)			
30	27 (3.4)	4 (1.2)	.04
60	40 (5.0)	8 (2.4)	.04
90	52 (6.5)	11 (3.2)	.03

ED visit rates demonstrated a similar pattern. At 30 days, ED visit rates were 3.4% (27/795) in the nonparticipating group and 1.2% (4/340) in the participating group (*P*=.04). At 60 days, visit rates were 5.0% (40/795) in the nonparticipating group and 2.4% (8/340) in the participating group (*P*=.04). At 90 days, visit rates were 6.5% (52/795) in the nonparticipating group and 3.2% (11/340) in the participating group (*P*=.03).

To further characterize postoperative health care utilization, an exploratory descriptive analysis was performed examining readmissions related to complications of the index sinonasal procedure. Among the 202 patients enrolled in the CareSense sinus surgery pathway, 14 (7%) experienced a readmission, compared with 7 (5%) of the 138 patients enrolled in the septoplasty pathway (*P*=.45). Of all readmissions in the participating group, 6 of 25 (24%) were related to postoperative complications of the index procedure, including 5 cases of uncontrolled epistaxis and 1 nasoseptal abscess. In the nonparticipating group, 21 of 125 readmissions (17%) were procedure-related (*P*=.41), most commonly due to uncontrolled epistaxis (18 cases), with additional causes including sinusitis flares requiring intravenous antibiotics (2 cases) and septal hematoma (1 case). When related to the index procedure, readmissions were predominantly attributable to uncontrolled epistaxis in both cohorts. Unrelated admissions included asthma exacerbations, chest pain, abdominal pain, and COVID-19–related illness.

The mean postoperative LOS among patients who were readmitted was 1.68 days in the nonparticipating group compared with 0.17 days in the participating group (*P*<.001).

### Multivariable Analysis

**Figure 1.**
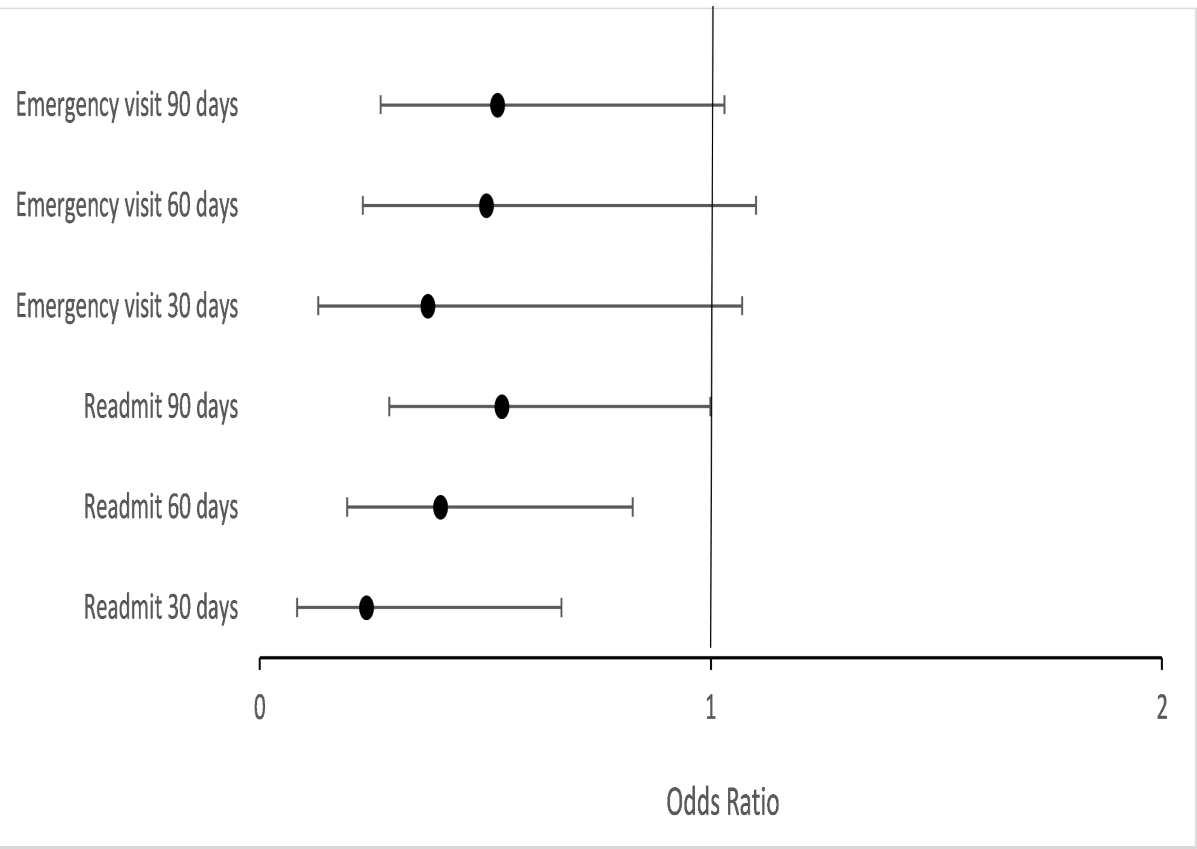
Forest plot illustrating the odds of readmissions and emergency visits between patients who were and were not enrolled in CareSense at 30, 60, and 90 days.

After adjusting for age, sex, hypertension, and diabetes, multivariable logistic regression demonstrated that CareSense participation was associated with lower odds of readmission at 30 days (OR 0.24, 95% CI 0.08‐0.75; *P*=.007), 60 days (OR 0.4, 95% CI 0.19‐0.83; *P*=.01), and 90 days (OR 0.54, 95% CI 0.29‐0.99; *P*=.04) when compared to the nonparticipating group (Figure 1). Although not statistically significant, CareSense participation trended toward an association with lower odds of emergency department visits at 30 days (OR 0.37, 95% CI 0.13‐1.07; *P*=.07), 60 days (OR 0.5, 95% CI 0.23‐1.1; *P*=.09), and 90 days (OR 0.53, 95% CI 0.27‐1.03; *P*=.06) when compared to the nonparticipating group (Figure 1).

## Discussion

The rapid expansion of mHealth technologies has raised important questions regarding their role in improving clinical outcomes and health care utilization. Prior studies have reported associations between mHealth interventions and improved outcomes across multiple medical and surgical domains, including chronic pulmonary disease, heart failure, diabetes, and orthopedic surgery [5,16,17]. These effects have been attributed to improved access to health information, enhanced communication between patients and care teams, and increased patient engagement, which may collectively support self-management and appropriate health care utilization [18,19]. However, evidence evaluating mHealth interventions in otolaryngologic surgery, particularly with respect to postoperative health care utilization, remains limited.

In this retrospective observational study, perioperative enrollment in CareSense was associated with lower rates of all-cause hospital readmissions and ED visits at 30, 60, and 90 days following sinonasal surgery, as well as shorter hospital LOS among patients who were readmitted. These findings suggest that the use of a perioperative patient engagement platform may be associated with reduced postoperative health care utilization in this population. From a health systems perspective, even modest reductions in readmissions and length of stay may have meaningful implications for cost and resource utilization, given that a single inpatient hospital day in the United States is estimated to cost between US $2000 and $3000 [20].

Notably, the majority of readmissions observed in both cohorts were unrelated to the index sinonasal procedure. This finding suggests that the observed associations may not reflect a direct effect on surgical complications, but rather broader differences in postoperative care-seeking behavior and health system utilization. Importantly, procedure-related readmissions were uncommon and were predominantly driven by postoperative epistaxis in both cohorts, suggesting that differences in overall health care utilization were not driven by an increase in surgery-specific complications. Enrollment in CareSense may serve as a proxy for patient engagement, with participating patients potentially more likely to manage symptoms proactively, adhere to discharge instructions, or seek outpatient guidance before presenting for acute care. The platform’s structured perioperative education and automated communication may also help establish expectations and provide reassurance during recovery, thereby reducing uncertainty-driven utilization of emergency services. One study of a perioperative mHealth intervention demonstrated reductions in postoperative care needs and improvements in patient self-management, even when effects on disease-specific outcomes were modest [21]. These findings support the hypothesis that digital perioperative engagement may influence health care utilization by addressing information gaps and postoperative uncertainty rather than directly preventing surgical complications. Collectively, these features may support patient confidence and self-efficacy before and after surgery, contributing to reduced reliance on acute care services in the postoperative period.

Baseline differences between participating and nonparticipating patients indicate that there should be cautious interpretation. Patients who enrolled in CareSense were younger and had lower rates of hypertension and diabetes, characteristics that may independently influence postoperative outcomes and health care utilization. Although multivariable adjustment was performed, residual confounding is likely in any observational study of voluntary technology adoption. The demographic profile of CareSense participants aligns with prior literature demonstrating greater uptake of digital health tools among younger patients [22,23]. Interestingly, a higher proportion of CareSense participants were male, which contrasts with prior studies reporting higher digital health engagement among younger women [22,23]. Additionally, nonparticipating patients had higher rates of chronic comorbidities such as hypertension and diabetes mellitus, which are known to adversely impact postoperative outcomes in several surgical populations, although an association with sinonasal surgery outcomes has not been well established [24-27]. Importantly, even after adjusting for these measured differences, CareSense participation remained associated with lower odds of postoperative readmission at multiple time points. Despite these differences, CareSense participation remained associated with lower odds of readmission across multiple time points after adjustment, consistent with prior reports of CareSense-associated benefits in orthopedic surgery populations [5].

This study has limitations. Selection bias is inherent to voluntary mHealth enrollment, and unmeasured factors such as health literacy, social support, and baseline health care engagement may have influenced the observed associations. Outcomes were limited to encounters captured within a single integrated health system, and healthcare utilization outside the system was not assessed. Procedure-related readmissions accounted for a small proportion of events, limiting the power to evaluate surgery-specific complications. Additionally, sinonasal disease–specific patient-reported outcome measures and endoscopic findings were unavailable, precluding assessment of symptom severity or disease control as mediators of postoperative utilization. Patients without access to a mobile phone or email address were not eligible for CareSense enrollment, raising the possibility that unmeasured social determinants of health contributed to group differences.

Despite these limitations, this study provides evidence from a large real-world cohort that perioperative enrollment in a patient-facing mHealth platform is associated with reduced postoperative health care utilization following sinonasal surgery. These findings are observational and hypothesis-generating, and they highlight the need for prospective multicenter studies to more definitively evaluate causal relationships with pre-specified subgroups, characterize engagement intensity, and identify which components of digital perioperative care are most effective. Future studies could prospectively assess subgroup effects by surgical procedure, baseline health status, and measured engagement metrics (eg, message opens or survey completion), to better understand which patients derive the greatest benefit from perioperative mHealth support. As mHealth platforms become increasingly integrated into routine surgical care, understanding their role in optimizing postoperative outcomes and health care utilization will be essential.

## Supplementary material

10.2196/75403 Multimedia Appendix 1 CareSense Sinus Surgery Pathway Content.

10.2196/75403 Multimedia Appendix 2 CareSense Septoplasty with Turbinate Reduction Pathway Content.

## Abbreviations

ED: Emergency Department
EHR: Electronic Health Record
GEE: generalized estimated equations
LOS: length of stay
mHealth: mobile health
