# Supplementary material for: Outcomes of Mobile Health Use in Sinonasal Surgery: Retrospective Cohort Study
Source: JMIR Mhealth Uhealth. 2026 Jul 21;14:e75403. doi: 10.2196/75403 (PMC13386657; doi:10.2196/75403)
Supplement: Multimedia Appendix 1 [file mhealth-v14-e75403-s001.docx]

CareSense Sinus Surgery Pathway Content

| **Anchor: Sign up**  **Day: 0**  **Time:**  **Group:**  **Attachments:**  Good day from Houston Methodist! Thank you for scheduling sinus surgery. We will help you get the most out of your surgery by sending you text messages and emails both before and after your surgery. We want to make sure you’re doing well and recovering just the way you hope. Plus, there is a lot of information to remember, and we want to spread the information out so that you don’t get overwhelmed. Please store us in your phone as “Houston Methodist ENT.” Messages are not secure and have some risk of disclosure to third parties. You can opt out of receiving text messages and emails at any time by texting the word “STOP” in response to any text. |
| --- |
| **Anchor: Sign up**  **Day: 0 - Day of Sign Up (text link) - 11:00 AM**  **Time:**  **Group:**  **Survey: Houston-Methodist-Email-Confirmation**  **Type: Text link**  We just would like to take a minute to confirm some of your contact information.   - No Email   - It looks like we don't have your email address on file. Would you like to provide it?     - Yes       1. What is your email address? - No - Has Email   - The following is the current email address we have on file for you: <patient’s email.> Would you like to update this information?     - Yes       - What is your email address?     - No - Thank you for your responses. |
| **Anchor: Sign up**  **Day: 0**  **Time:**  **Group:** Dr. Takashima or Dr. Ahmed  **Attachments:**  Subject: Welcome to Houston Methodist ENT Care  Body:  Welcome to Houston Methodist. You have chosen one of the nation’s leading academic medical centers for your ENT care. Houston Methodist Hospital, our flagship academic hospital in the Texas Medical Center, is consistently ranked as the No. 1 hospital in Texas and one of America’s “Best Hospitals” by *U.S. News & World Report.*  We at the Houston Methodist Sinus Center are committed to providing you with the highest level of care in a comfortable and caring environment. We want you to have as complete of an understanding as possible about your sinus condition and about our recommendations for treatment. Please feel free to ask questions about any aspect of your care; we will be happy to make sure that all of your questions are answered.  We are contacting you through this method because your preferences indicate that you are comfortable receiving communication from Houston Methodist. Please be mindful that texts and emails are not secure and pose some risks for disclosure, and if you are using this method of communication, you are accepting these risks. For secure communication, please enroll in the patient portal (sign up link in footer of emails).    Below are some common questions we hear from patients. We hope you find our answers to be helpful! Later, we will send you a detailed email on what to expect immediately before, during, and after your procedure.  Chronic sinusitis is an illness characterized by prolonged infection and inflammation of the lining of the nose and the sinuses. Patients with chronic sinusitis experience a variety of symptoms, including facial pressure, nasal congestion, discolored nasal discharge, and/or “post-nasal drip,” which do not clear up with medication. After reviewing your medical history and x-ray studies, we have determined you will benefit from surgery.  **Question: Who is performing my surgery?**  **Answer**: Dr. Mas Takashima will be performing your surgery. He has over 15 years of experience performing endoscopic sinus surgery and is considered one of the nation’s leading experts. He is fellowship-trained in performing advanced endoscopic sinus surgery and has achieved outstanding results for his patients.    **Answer**: Dr. Omar Ahmed will be performing your surgery. He is fellowship-trained in performing advanced endoscopic sinus surgery. He trained at Johns Hopkins Hospital under the world’s leading experts, bringing his expertise here to Houston Methodist Hospital.  **Question: What is endoscopic sinus surgery?**  **Answer:** Endoscopic sinus surgery is a procedure where we open the natural drainage pathways of the sinuses to restore your function and health. In chronic sinusitis, the sinuses are unable to drain adequately due to inflammation of the drainage pathways. As a result, nasal secretions become trapped in the sinuses and become infected often.    The goal of surgery is to carefully remove the thin, delicate bone and mucous membranes that block the drainage pathways of the sinuses. The term “endoscopic” refers to the use of small fiber optic telescopes that allow the surgery to be performed through the nostrils, without the need for any skin incisions. Endoscopic sinus surgery is generally performed on an outpatient basis.    **Question: What are the complications/risks of surgery?**  **Answer:** As with any surgical procedure, endoscopic sinus surgery has associated risks. Although the chances of a complication occurring are very small, it is important that you understand the potential complications and ask your surgeon about any concerns you may have.   - Bleeding: Most sinus surgeries involve some degree of blood loss. However, on occasion, significant bleeding may require that we stop the procedure. Blood transfusion is rarely necessary and is only given if your health would be compromised without it. - Recurrence of disease: Although endoscopic sinus surgery provides benefits for most patients and helps relieve their symptoms, surgery is not a cure for sinusitis. Therefore, you should expect to continue with your sinus medications even after successful sinus surgery, although you can expect to take less medications. In some cases, additional “touch-up” surgery may be necessary. - Spinal fluid leak: Because the sinuses are close to the brain, there is a rare chance of creating a leak of spinal fluid (the fluid lining the brain) or injuring the brain. Should the rare complication of a spinal fluid leak occur, it may create a potential pathway for infection, which could result in meningitis. If a spinal fluid leak were to occur, it may require surgical closure and extend your hospitalization. - Visual problems: There have been a few reports of vision loss after sinus surgery. The potential for recovery in such cases is not good. Fortunately, such a complication is extremely rare. Double vision has also been reported following sinus surgery. Persistent tearing of the eye can also result from surgery, but this problem usually resolves on its own. - Other uncommon risks of surgery include alteration of sense of smell or taste; persistence and/or worsening of sinus symptoms and facial pain; and swelling or bruising of the area around the eye. Some patients have a deviation of the nasal septum that needs to be corrected at the time of surgery through a short procedure called septoplasty. If you require septoplasty, there are additional risks associated with this procedure. |
| **Anchor: Sign up**  **Day: 0**  **Time:**  **Group: All doctors EXCEPT Dr. Takashima or Dr. Ahmed**  **Attachments:**  Subject: Welcome to Houston Methodist ENT Care  Body:  Welcome to Houston Methodist. You have chosen one of the nation’s leading academic medical centers for your ENT care. Houston Methodist Hospital, our flagship academic hospital in the Texas Medical Center, is consistently ranked as the No. 1 hospital in Texas and one of America’s “Best Hospitals” by *U.S. News & World Report.*  We at the Houston Methodist Sinus Center are committed to providing you with the highest level of care in a comfortable and caring environment. We want you to have as complete of an understanding as possible about your sinus condition and about our recommendations for treatment. Please feel free to ask questions about any aspect of your care; we will be happy to make sure that all of your questions are answered.  We are contacting you through this method because your preferences indicate that you are comfortable receiving communication from Houston Methodist. Please be mindful that texts and emails are not secure and pose some risks for disclosure, and if you are using this method of communication, you are accepting these risks. For secure communication, please enroll in the patient portal (sign up link in footer of emails).    Below are some common questions we hear from patients. We hope you find our answers to be helpful! Later, we will send you a detailed email on what to expect immediately before, during, and after your procedure.  Chronic sinusitis is an illness characterized by prolonged infection and inflammation of  the lining of the nose and the sinuses. Patients with chronic sinusitis experience a  variety of symptoms, including facial pressure, nasal congestion, discolored nasal discharge, and/or “post-nasal drip,” which do not clear up with medication. After reviewing your medical history and x-ray studies, we have determined you will benefit from surgery.  **Question: What is endoscopic sinus surgery?**  **Answer:** Endoscopic sinus surgery is a procedure where we open the natural drainage pathways of the sinuses to restore your function and health. In chronic sinusitis, the sinuses are unable to drain adequately due to inflammation of the drainage pathways. As a result, nasal secretions become trapped in the sinuses and become infected often.    The goal of surgery is to carefully remove the thin, delicate bone and mucous membranes that block the drainage pathways of the sinuses. The term “endoscopic” refers to the use of small fiber optic telescopes that allow the surgery to be performed through the nostrils, without the need for any skin incisions. Endoscopic sinus surgery is generally performed on an outpatient basis.    **Question: What are the complications/risks of surgery?**  **Answer:** As with any surgical procedure, endoscopic sinus surgery has associated risks. Although the chances of a complication occurring are very small, it is important that you understand the potential complications and ask your surgeon about any concerns you may have.   - Bleeding: Most sinus surgeries involve some degree of blood loss. However, on occasion, significant bleeding may require that we stop the procedure. Blood transfusion is rarely necessary and is only given if your health would be compromised without it. - Recurrence of disease: Although endoscopic sinus surgery provides benefits for most patients and helps relieve their symptoms, surgery is not a cure for sinusitis. Therefore, you should expect to continue with your sinus medications even after successful sinus surgery, although you can expect to take less medications. In some cases, additional “touch-up” surgery may be necessary. - Spinal fluid leak: Because the sinuses are close to the brain, there is a rare chance of creating a leak of spinal fluid (the fluid lining the brain) or injuring the brain. Should the rare complication of a spinal fluid leak occur, it may create a potential pathway for infection, which could result in meningitis. If a spinal fluid leak were to occur, it may require surgical closure and extend your hospitalization. - Visual problems: There have been a few reports of vision loss after sinus surgery. The potential for recovery in such cases is not good. Fortunately, such a complication is extremely rare. Double vision has also been reported following sinus surgery. Persistent tearing of the eye can also result from surgery, but this problem usually resolves on its own. - Other uncommon risks of surgery include alteration of sense of smell or taste; persistence and/or worsening of sinus symptoms and facial pain; and swelling or bruising of the area around the eye. Some patients have a deviation of the nasal septum that needs to be corrected at the time of surgery through a short procedure called septoplasty. If you require septoplasty, there are additional risks associated with this procedure. |
| **Anchor: Day of surgery**  **Day: -25**  **Time:**  **Group:**  **Attachments:**  Good day from Houston Methodist! If you smoke, it is critical that you stop smoking for at least three weeks prior to your surgery, and that you do not smoke for at least four weeks after surgery.  Smoking during this critical window can seriously interfere with the success of the operation, resulting in excessive scarring and/or potential failure of the operation. Your primary care physician can help direct you to resources to assist with smoking cessation. |
| **Anchor: Day of surgery**  **Day: -20**  **Time:**  **Group:**  **Attachments:**  Subject: Taking your Medications: Houston Methodist  Body:  In preparation for your surgery, we may prescribe medications for you to get your sinuses ready. The medications may include antibiotics and/or oral steroids. If we determine that you should be on medications, please be sure to start the medications on the appropriate day and to follow the instructions closely.    You should also avoid taking the following medications for at least fourteen days prior to surgery. We will remind you again at that time.   - Aspirin - Ibuprofen (Motrin/Advil) - Naproxen (Aleve) - Other non-steroidal anti-inflammatories (NSAIDs) - Vitamin E (a multivitamin is ok) - Fish oil - Ginkgo biloba - Garlic (tablets) - Ginseng   Reason for stopping them: These medications can thin the blood and create excessive bleeding both during surgery and right after surgery. Tylenol is safe and may be taken anytime up to the day of your surgery.  St. John’s wort should also be avoided for two weeks prior to surgery because of possible interactions with anesthesia medications. |
| **Anchor: Day of surgery**  **Day: -14**  **Time:**  **Group:**  **Attachments:**  Houston Methodist here! Remember, you should avoid taking the following medications for at least  fourteen days prior to your surgery.   - Aspirin - Ibuprofen (Motrin/Advil) - Naproxen (Aleve) - Other non-steroidal anti-inflammatories (NSAIDs) - Vitamin E (a multivitamin is ok) - Fish oil - Ginkgo biloba - Garlic (tablets) - Ginseng - St. John’s wort   Second, it is important that you inform your primary care physician that you are planning to have sinus surgery. We will make every effort to keep your primary care physician informed regarding your medical status both before and after your surgery. |
| **Anchor: Day of Surgery**  **Day: -14**  **SurveyID: ENT Pathways Confirm Surgery Mapping**  **Type: SMS/Monitoring Survey**  Good day from Houston Methodist. Your procedure is scheduled two weeks from today. Given that there is currently a long waitlist for surgeries, please confirm this date still works for you.   - Press 1 to Confirm - Press 2 to Reschedule   - Your physician’s office has been notified and will be in contact with you shortly to reschedule your procedure.   - [Generate CN Alert] "Patient has indicated that they would like to reschedule their procedure." |
| **Anchor: Day of surgery**  **Day: -10**  **Time:**  **Group:**  **Attachments:**  *Text:* Friendly reminder from Houston Methodist: You can always opt out of messages by writing “STOP” in response to our text messages.  *Call:* " Friendly reminder from Houston Methodist: You can always opt out of messages by writing “STOP” in response to our emails or if you are not receiving emails, by calling your physician's office and asking to be taken off the system.” |
| **~~Anchor: Day of surgery~~**  **~~Day: -4~~**  **~~Time:~~**  **~~Group:~~**  **~~Attachments:~~**  ~~Good day from Houston Methodist! If your ENT prescribed antibiotics and/or steroids for you to start taking prior to surgery, you should start them now.~~ |
| **Anchor: Day of surgery**  **Day: -1**  **Time: 11 am**  **Group:**  **Attachments:**  Good day from Houston Methodist! You are scheduled for a sinus surgery tomorrow! If you are on blood thinners and have not spoken with your physician regarding when and how to stop your medications, please call your physician’s office (the one who prescribed the medicine).    For your procedure tomorrow, go to the Houston Methodist Hospital Outpatient Center on the 18^th^ floor. You can also register and check in on the 18^th^ floor. Park in the Outpatient Center Garage. Please bring your medicine list and your day’s supply of medicines with you to the procedure. Also, make sure to arrange for a ride to take you home after your procedure. |
| **Anchor: Surgery**  **Day: -1**  **Role: Care Partner**  Subject: Belongings in Hospital  Things your loved one need while they are in the hospital:  Photo ID  Insurance Card  Credit Card (If payment hasn’t been made using MyChart)  Prescription Glasses  Hearing Aids  Cell phones  Please take the following items home with you while your loved one is in the hospital:  Purses  Wallets  Laptops  iPads  Necklaces  Watches  Electric Appliances  Large Suitcases  Sentimental items such as pillows, blankets, sweaters, or anything of value that cannot be replaced. If your loved one forgets and brings these items, please take them back home with you. |
| **Anchor: Surgery**  **Day: -1**  Subject: Belongings in Hospital  Things you need while you are in the hospital:  Photo ID  Insurance Card  Credit Card (If you have not paid using MyChart)  Please give these items to your family members before any procedures. If your family member is not present, please let someone know you are leaving these items in your room.  Prescription Glasses  Hearing Aids  Cell phones -Please place your cell phone on the bedside table during your stay and not in the bed or on the stretcher during transportation.  Please send the following items home with your family during your hospital stay:  Purses  Wallets  Laptops  iPads  Necklaces  Watches  Electric Appliances  Large Suitcases  Sentimental items such as pillows, blankets, sweaters, or anything of value that cannot be replaced. If you forget and bring these items, please send them back home with your family immediately.  NOTE: The best person to keep your personal belongings is your family member. Thank you for securing your personal belongings by leaving them at home or giving them to your family member. |
| **Date of Surgery=No Content Sent to Patient** |
| **Anchor: Day of surgery**  **Day: +1**  **Time:**  **Group:**  **Attachments:**  A heartfelt congratulations from Houston Methodist on completing your surgery! Don’t forget to start using your sinus rinse kits (NeilMed Sinus Rinse Kit). These must be used at least three times each day. Your doctor or nurse will show you how to perform the irrigations. You can expect some bloody discharge with the rinses for the first few days. These rinses are critical for success! Continue with your sinus rinse kits until we tell you that you can stop. You should also start the antibiotics prescribed by your ENT.    Please check your email today for an outline of what to expect and what should be points of concern during your recovery. We also gave you some tips on how to feel your best! |
| **Anchor: Day of surgery**  **Day: +1**  **Time:**  **Group:**  **Attachments:**  Subject: What to Expect After Surgery: Houston Methodist  Body:  Below is an outline of what to expect and what should be points of concern during your recovery. We also gave you some tips on how to feel your best!    **What can I expect after endoscopic sinus surgery?**    Bleeding   - **What to expect?** It is normal to have some bleeding after sinus surgery. You can expect some bloody discharge for the first three to five days after surgery, especially after you irrigate your sinuses. - **What can you do about it?** If steady bleeding occurs after surgery, tilt your head back slightly and breathe through your nose gently. You may dab your nose with tissue, but avoid any nose blowing. If this does not stop the bleeding, you may use Afrin nasal spray. Several sprays will usually stop any bleeding. If Afrin fails to stop steady nasal bleeding, you should call our office or the on-call doctor at 713-441-1368.   Pain   - **What to expect?** You should expect some nasal and sinus pressure and pain for the first several days after surgery. This may feel like a sinus infection or a dull ache in your sinuses. Throat discomfort related to the breathing tube placed by Anesthesia during surgery is also common. - **What can you do about it?** You can use Tylenol for mild to moderate pain every four to six hours. If you have severe pain, you can also use the narcotic pain medication prescribed (i.e. typically Tylenol with codeine), but we would reserve this for severe pain. Narcotic pain medications have several unpleasant side effects (like constipation), so we would suggest using it only when your pain is severe. If you prefer a non-narcotic medication, extra-strength Tylenol is safe and works well. You should avoid aspirin and NSAIDs such as Motrin, Advil, and Aleve.     Fatigue   - **What to expect?** You can expect to feel very tired for the first week after surgery. This is normal. - **What can you do about it?** Many patients plan on taking at least one week off work to recover. Every patient is different and some return to work sooner.   Nasal congestion and discharge   - **What to expect?** You will have nasal congestion and discharge for the first few weeks after surgery. - **What can you do about it?** Your nasal passage and breathing should return to normal two to three weeks after surgery. During your visits with us after surgery, we will clean your nose and sinuses of fluid and blood left behind after surgery. There will be some discomfort involved with the cleaning, so it is best to take a pain medication 45 minutes before your visit.   **When should I call after surgery, because there might be a problem in my recovery?**  Call us at 713-441-1368 if you experience any of the following:   - Fever after the day of surgery higher than 101 degrees - Steady, brisk nose bleeding that doesn’t get better after using Afrin - Sudden visual changes or eye swelling - Severe headache or neck stiffness - Severe diarrhea - Constant clear watery discharge after the first week of surgery     **Who should I call?**   - During the day, you should call the clinic at 713-441-1368. - After hours, you should call 713-441-1368 and your doctor will be paged.   **What postoperative appointments will I have?**   - Your first visit will likely be one week after surgery. The goal of this visit is to see how you are doing and to clean out your sinuses. There will be some discomfort involved with the cleaning, so it is best to take a pain medication 45 minutes before your visit. - Your second visit will be two weeks after surgery. The goal of this visit is to remove crusting and check to see how your sinuses are healing. - You may have another visit six weeks after surgery. The goal of this visit will be a check-up of your sinuses to make sure things have healed appropriately. It is essential that you return for all scheduled follow-up appointments, as careful postoperative care is critical to the success of your surgery. - After three to four months, most patients’ sinuses have healed completely, and visits for maintenance care are then made a few times per year. |
| **Anchor: Day of Surgery**  **Day: +3**  **Time:**  **Group:**  **Attachments:**  Good day from Houston Methodist. You should not blow your nose for the first week following your surgery. A saline spray may be used several times per day to relieve nasal irritation. You should not perform exercises or any other exertional activity for at least two weeks following surgery. This includes no bending, lifting more than about 10 lbs, or straining. Your surgeon will be able to advise you when it is safe to begin exercising again. |
| **Anchor: Day of Surgery**  **Day: +5**  **Time:**  **Group:**  **Survey: Houston-Sinus-Symptoms-Email**  **Type: Text link**  Houston Methodist here! Just checking in on you.  Are you experiencing any of the following symptoms?  o Fever after the day of surgery higher than 101 degrees  o Steady, brisk nose bleeding that doesn’t get better after using Afrin  o Sudden visual changes or eye swelling  o Severe headache or neck stiffness  o Severe diarrhea  o Constant clear watery discharge after the first week of surgery     - Yes   - Please call us at 713-441-1368. - No   - Good to hear! Don’t forget to schedule your follow-up appointment for your first week after surgery! |
| **Anchor: Surgery**  **Day: +11**  **Time:**  **Group:**  **Survey: Houston-Sinus-Satisfaction**  **Type: Text link**  Good day from Houston Methodist. We will be stopping our messages soon, because you’re on the road to recovery! We’re hoping you can answer a few questions about our system.  How much do you agree or disagree with the following statement?  • “It was helpful to me to receive alerts, reminders, and emails from this program before and after my surgery.”  1. Strongly agree  2. Agree  3. Undecided  4. Disagree  5. Strongly disagree  Please explain why you chose this option. What did you like or not like about the system? (Free text space)  How much do you agree or disagree with the following statement?  • “This program improved my overall knowledge about my surgery and recovery compared to what I knew before.”  1. Strongly agree  2. Agree  3. Undecided  4. Disagree  5. Strongly disagree  Please explain why you chose this option. How did this system improve or not improve your knowledge? (Free text space) |
| **Anchor: Surgery**  **Day: +35**  **Time:**  **Group:**  **Attachments:**  Houston Methodist here! You should now be well on your way to recovery and you will no longer receive messages from this system. If you have any questions or concerns as you continue to recover, please follow up with your physician. Take care. |
