# Supplementary material for: Outcomes of Mobile Health Use in Sinonasal Surgery: Retrospective Cohort Study
Source: JMIR Mhealth Uhealth. 2026 Jul 21;14:e75403. doi: 10.2196/75403 (PMC13386657; doi:10.2196/75403)
Supplement: Multimedia Appendix 2 [file mhealth-v14-e75403-s002.docx]

CareSense Septoplasty with Turbinate Reduction Pathway Content

| **Anchor: Sign up**  **Day: 0**  **Time:**  **Group:**  **Attachments:**  **Repeats:**  Hello from Houston Methodist! Thank you for scheduling your septal and turbinate surgery with us. We will help you get the most out of your surgery by sending you information both before and after your surgery. We want to make sure you’re doing well and recovering just the way you hope. There is a lot of information to remember, so we will spread the information out so that you don’t get overwhelmed.    Please store us in your phone as “Houston Methodist ENT.” Messages are not secure and have some risk of disclosure to third parties. You can opt out of receiving information at any time by responding “STOP” to any text. |
| --- |
| **Anchor: Sign up**  **Day: 0**  **Time:**  **Group:**  **Attachments:**  Subject: Welcome to Houston Methodist ENT  Body:  Welcome to Houston Methodist. You have chosen one of the nation’s leading academic medical centers for your ENT care. Houston Methodist Hospital, our flagship academic hospital in the Texas Medical Center, consistently ranks as the No. 1 hospital in Texas and one of America’s “Best Hospitals” by *U.S. News & World Report.*  We are contacting you through this method because your preferences indicate that you are comfortable receiving communication from Houston Methodist. Please be mindful that this method of communication is not secure and poses some risks for disclosure, and by continuing, you are accepting these risks.    Below are some common questions we hear from patients. We hope you find our answers helpful! Later, we will send you some detailed information on what to expect immediately before, during, and after your procedure. We at Houston Methodist ENT are committed to providing you with the highest level of care in a comfortable and caring environment. We want you to have as complete of an understanding about your surgery and our recommendations for treatment as possible. Please feel free to ask questions about any aspect of your care; we will be happy to make sure that all of your questions have been answered.    **Question: Who will be performing my surgery?**  **Answer:** Dr. Mas Takashima will be performing your surgery. He has over 15 years of experience performing sinus and nasal surgery and is considered one of the nation’s leading experts in sinus and nasal surgery. He is fellowship-trained in performing advanced endoscopic sinus and nasal surgery and has achieved outstanding results for his patients.    **Answer:** Dr. Omar Ahmed will be performing your surgery. He is fellowship-trained in performing advanced sinus and nasal surgery. He trained at Johns Hopkins Hospital under the world’s leading experts before bringing his expertise here to Houston Methodist Hospital.    **Question: What is a nasal or deviated septum and how does that contribute to my symptoms?**  **Answer:** A deviated septum occurs when your nasal septum — the thin wall of cartilage or bone that separates your right and left nasal passages — is moved to one side.    **Question: How does a deviated septum develop?**  **Answer:** A deviated septum can be caused by several conditions:   - A deviated septum can be present at birth or can occur over time as the face and nose grow. - Deviated septums are often caused by nasal injuries that cause the nasal septum to be moved out of place.     **Question: What kind of symptoms does a deviated septum cause?**  **Answer:** The most common sign or symptom of a deviated septum is difficulty breathing out your nose. In some cases, a deviated septum can also contribute to sinus infections, nose bleeds, and headaches. In severe cases, a deviated septum can make the nose look twisted from the outside. After reviewing your medical history and examining both the inside and outside of your nose, we have determined you would benefit from nasal septal surgery, also known as a septoplasty.    **Question: What is nasal septal surgery/septoplasty?**  **Answer:** A septoplasty is the usual way to repair a deviated septum. During septoplasty surgery, your nasal septum is straightened and repositioned in the center of your nose. This may require your surgeon to reposition or remove parts of your septum in order to open your airway.    The level of improvement you can expect from surgery depends on the severity of your deviation. Symptoms caused by the deviated septum — particularly nasal blockage — may completely go away. The main goal of nasal septal surgery is to improve your breathing. Any other nasal or sinus conditions you have that affect the tissues lining your nose (such as allergies) can't be cured with only surgery.    **Question: What are turbinates and why are they causing problems?**  **Answer:** Turbinates are small structures within your nose that cleanse and humidify air as it passes through your nostrils and into your lungs. These can sometimes increase or enlarge and cause nasal obstruction or blockage. Enlarged turbinates in combination with a deviated septum can cause nasal obstruction. Your ENT believes that reducing your turbinates in addition to fixing your deviated septum will help to fix your nasal obstruction.    **Question: How is a turbinate reduction performed?**  **Answer:** This surgery is performed with your septoplasty while you are under general anesthesia. Depending on the size of the turbinates, there are different techniques that may be used to reduce turbinates. If your turbinates are very large, the bottom 1/3 of your turbinates will be removed. If they are moderately large, some soft tissue in the middle of the turbinate will be reduced or shrunk. This does not add any extra recovery time and can greatly improve your breathing.    **Question: What are the complications/risks of surgery?**  **Answer:** As with any surgical procedure, septal surgery has risks. Although the chances of a problem occurring are very small, it is important that you understand the potential risks and speak with your surgeon about any concerns you may have.   - Persistent symptoms of nasal obstruction: On occasion, patients continue to have some symptoms of nasal obstruction or difficulty breathing. This can result in allergies or irritation to the nasal lining. It can also lead to continued problems or narrowing of the nasal airway. - Nasal scarring: On rare occasions, scarring within the nasal cavity can occur. This can affect the nasal airway and make it difficult to breathe. If this occurs, the scars can be treated. - Septal perforation: On rare occasions, the blood supply to the nasal septum can be reduced. This can result in a hole in the septum that may crust or bleed. In most cases, if a perforation (or hole) occurs, it can be repaired. |
| **Anchor: Sign up**  **Day: +5**  **Time:**  **Group:**  **Attachments:**  Subject: Expectations for Before, During, and After Surgery: Houston Methodist  Here’s what you can expect before, during, and after surgery. We at the Houston Methodist ENT Surgery Center are committed to providing you with the highest level of care in a comfortable and caring environment. We want you to have as complete of an understanding about your nasal condition and our recommendations for treatment as possible. Please feel free to ask questions about any aspect of your care; we will be happy to make sure that all of your questions have been answered.    **BEFORE SURGERY**    Taking your medications: We may start a nasal hygiene routine for you in order to get your nose ready for surgery. The routine may include nasal saline irrigations (wash outs) or medications, like antihistamines, antibiotics, and/or oral steroids. If we determine that you should be on medications, please be sure to start the medications on the appropriate day and to closely follow the instructions.    In addition, you should avoid taking the following medications for at least 14 days prior to surgery (we will remind you again at that time):   - Aspirin - Ibuprofen (Motrin/Advil) - Naproxen (Aleve) - Other non-steroidal anti-inflammatories (NSAIDs) - Vitamin E (multivitamin is ok) - Fish oil - Ginkgo biloba - Garlic (tablets) - Ginseng     You should stop taking these medications because they can thin the blood and create excessive bleeding both during surgery and right after surgery. Tylenol is safe and may be taken any time up  to the day of surgery. St. John’s wort should also be avoided for two weeks prior to surgery because of possible interactions with anesthesia medications.  Smoking: If you smoke, it is critical that you stop smoking for at least three weeks prior to surgery, and at least four weeks after surgery. Smoking during this critical time frame can seriously interfere with the success of the operation, resulting in excessive scarring and/or the operation may fail. Your primary care physician can direct you to resources that may assist you with smoking cessation.    Talking with your primary care physician: It is important that you tell your primary care physician that you are planning to have septal surgery. Your primary care physician can be of great assistance in helping to make sure that you are medically cleared for surgery. We will make every effort to keep your primary care physician informed regarding your medical status both before and after your surgery.    Arranging for someone to take you home: You will need someone to pick you up after surgery. You should arrange for this ahead of time. You will not be allowed to drive yourself home after surgery.    **DURING SURGERY**     - In most cases, patients receive general anesthesia for this surgery. With a general anesthetic, you will be asleep for the entire procedure. - After your surgery is completed, you will spend about one hour in the Recovery Room followed by an additional recovery period of one to two hours in the Short Stay Unit. - Most patients feel well enough to go home the day of surgery. Some patients may require a one-night stay in the hospital if they need additional recovery time or have other medical problems that require special medical attention. You will most likely go home with gauze in your nose to absorb blood/other fluids after surgery.   **AFTER SURGERY**    One day after surgery onward: You will start nasal saline or nasal irrigations (washouts) the evening of your septoplasty and turbinate surgery. These should be performed at least two times each day. Your doctor or nurse will show you how to perform the irrigations before you leave the hospital. At first, they will feel strange if you haven’t done them before. Soon, however, they will become quite soothing, as they clean out any mucous or dried blood left in your nasal cavity. You can expect some bloody discharge with the irrigations for the first few days after surgery.    One week after surgery:    **Restrictions:**   - You should not blow your nose for the first week following surgery. A saline spray may be used several times each day to relieve nasal irritation. - You should not perform exercises or any other heavy activities for at least three weeks following surgery. This includes no bending, lifting more than about 8 pounds (about the weight of a gallon of milk), or straining. Your surgeon will be able to advise you when it is safe to begin exercising again. - You should plan on taking one week off from work to recover from surgery.     **Visits:**   - Your first visit with your ENT surgeon will likely be one week after surgery. The goals of this visit will be to see how you are doing, remove your nasal dressings, and clean out your nasal cavity. There may be crusting and old blood that gathers in your nasal cavity following the septoplasty. This will be removed with gentle suctioning during your initial postoperative visit.     Four weeks after surgery: Your second visit will be four weeks after surgery. The goals of this visit will be to evaluate your healing and remove any additional crusting that may have gathered within your nasal cavity.    8-12 weeks after surgery: You may have another visit anywhere from 8 to 12 weeks after your surgery. The goal of this visit will be to check your nasal cavity to make sure things have healed appropriately.    It is essential that you return for all scheduled follow-up appointments, as careful postoperative care is critical to the success of your surgery. |
| **Anchor: Surgery**  **Day: -14**  **SurveyID: ENT Pathways Confirm Surgery Mapping**  **Type: SMS/Monitoring Survey**  Good day from Houston Methodist. Your procedure is scheduled two weeks from today. Given that there is currently a long waitlist for surgeries, please confirm this date still works for you.   - Press 1 to Confirm - Press 2 to Reschedule   - Your physician’s office has been notified and will be in contact with you shortly to reschedule your procedure.   - [Generate CN Alert] "Patient has indicated that they would like to reschedule their procedure." |
| **Anchor: Surgery**  **Day: -4**  **Time:**  **Group:**  **Attachments:**  Good day from Houston Methodist! If your ENT surgeon has prescribed antibiotics and/or steroids to start taking prior to surgery, you should start them now.    At this point, you should have also arranged for a ride to take you home after surgery. You will not be allowed to drive yourself home. |
| **Anchor: Surgery**  **Day: -1**  **Time:**  **Group:**  **Attachments:**  Good day from Houston Methodist! You are scheduled for a septal and turbinate surgery tomorrow! If you are on blood thinners and have not spoken with your physician regarding when and how to stop your medications, please call your physician’s office (the one who prescribed the medicine).    For your procedure tomorrow, go to Houston Methodist Hospital Outpatient Center on the 18^th^ floor. You can also register and check in on the 18^th^ floor. Park in the Outpatient Center Garage.    Please bring your medicine list and your day’s supply of medicines with you to the procedure. Also, you should have arranged for a ride to take you home after surgery. |
| **Anchor: Surgery**  **Day: -1**  **Role: Care Partner**  Subject: Belongings in Hospital  Things your loved one needs while they are in the hospital:  Photo ID  Insurance Card  Credit Card (If payment hasn’t been made using MyChart)  Prescription Glasses  Hearing Aids  Cell phones  Please take the following items home with you while your loved one is in the hospital:  Purses  Wallets  Laptops  iPads  Necklaces  Watches  Electric Appliances  Large Suitcases  Sentimental items such as pillows, blankets, sweaters, or anything of value that cannot be replaced. If your loved one forgets and brings these items, please take them back home with you. |
| **Anchor: Surgery**  **Day: -1**  Subject: Belongings in Hospital  Things you need while you are in the hospital:  Photo ID  Insurance Card  Credit Card (If you have not paid using MyChart)  Please give these items to your family members before any procedures. If your family member is not present, please let someone know you are leaving these items in your room.  Prescription Glasses  Hearing Aids  Cell phones -Please place your cell phone on the bedside table during your stay and not in the bed or on the stretcher during transportation.  Please send the following items home with your family during your hospital stay:  Purses  Wallets  Laptops  iPads  Necklaces  Watches  Electric Appliances  Large Suitcases  Sentimental items such as pillows, blankets, sweaters, or anything of value that cannot be replaced. If you forget and bring these items, please send them back home with your family immediately.  NOTE: The best person to keep your personal belongings is your family member. Thank you for securing your personal belongings by leaving them at home or giving them to your family member. |
| **DAY OF SURGERY** |
| **Anchor: Surgery**  **Day: +2**  **Time:**  **Group:**  **Attachments:**  Congratulations from Houston Methodist on completing your surgery! Don’t forget to start your nasal rinses. These should be performed at least twice daily. Your doctor or nurse will show you how to perform the irrigations. You can expect some bloody discharge with the rinses for the first few days. Continue your nasal rinses for the first week after surgery. You should also start the antibiotics and pain medicine as prescribed by your ENT surgeon.    We just sent you some information on what to expect and look out for during your recovery — what is normal and what should be considered concerning. We hope you find this information provides some reassurance and you find it helpful! |
| **Anchor: Surgery**  **Day: +2**  **Time:**  **Group:**  **Attachments:**  Congratulations from Houston Methodist on completing your surgery! Don’t forget to start your nasal rinses. These should be performed at least twice daily. Your doctor or nurse will show you how to perform the irrigations. You can expect some bloody discharge with the rinses for the first few days. Continue your nasal rinses for the first week after surgery. You should also start the antibiotics and pain medicine as prescribed by your ENT surgeon.    Below is an outline of what to expect and what should be points of concern during your recovery. We also gave you some tips on how to feel your best!    What can I expect after septal and turbinate surgery?    **Bleeding:**   - What to expect? It is normal to have some bleeding after septal surgery. You can expect some bloody discharge for the first three to five days after surgery, especially after you rinse your sinuses. - What can you do about it? If steady bleeding occurs after surgery, tilt your head back slightly and breathe through your nose gently. You may dab your nose with a tissue but avoid any nose blowing. If this does not stop the bleeding, you may use Afrin nasal spray. Several sprays will usually stop any bleeding. If Afrin fails to stop steady nasal bleeding, you should call our office.     **Pain:**   - What to expect? You should expect some nasal and sinus pressure and pain for the first several days after surgery. This can feel like a dull ache in your nose and sinuses. Throat discomfort is also common; this is related to the breathing tube placed by anesthesia during surgery. You may also feel some numbness in your front teeth. This numbness is temporary and will go away. - What can you do about it? You can use Tylenol for mild to moderate pain every four to six hours. If you have severe pain, you can also use the Tylenol with codeine, but we recommend holding off on this for only severe pain. Narcotic pain medications have several unpleasant side effects (like constipation), so we suggest using them only when your pain is severe. If you prefer a non-narcotic medication, extra-strength Tylenol is safe and works well. You should avoid aspirin and NSAIDs such as Motrin, Advil, and Aleve.     **Fatigue:**   - What to expect? You can expect to feel very tired for the first several days to a week after surgery. This is normal. - What can you do about it? Many patients plan on taking at least one week off of work to recover. Every patient is different and some return to work sooner.     **Nasal congestion and discharge:**   - What to expect? You will have nasal congestion and discharge for the first few weeks after surgery. - What can you do about it? Your nasal passage and breathing should return to normal two to three weeks after surgery. During your visits with us after surgery, we will clean your nose and remove any mucous or dried blood that may have accumulated in your nasal cavity.     **When should I call after surgery because there might be a problem in my recovery?**    Call us if you experience any of the following:   - Fever after the day of surgery higher than 101 degrees F - Steady, brisk nose bleeding that doesn’t get better after using Afrin - Severe diarrhea - Nasal pain that does not respond to medication     Who should I call?   - During the day, you should call the clinic at 713-441-1368. - After hours, you should call 713-441-1368. |
| **Anchor: Surgery**  **Day: +4**  Houston Methodist here! Just checking in on you. Are you experiencing any of the following:    o Fever after the day of surgery higher than 101 degrees F  o Steady, brisk nose bleeding that doesn’t get better after using Afrin  o Severe diarrhea  o Severe nasal pain    Yes— Please call us at 713-441-1368.  No— That’s good to hear! Enjoy the rest of your day! |
| **Anchor: Surgery**  **Day: +7**  It’s Houston Methodist here! We will stop sending you messages now that you have undergone your surgery, but we’d love to get your feedback on what we did well and what we could have done better.    How much do you agree with the following statement: “I had a good surgical experience at Houston Methodist Outpatient Center.”     - Strongly agree - Agree - Undecided - Disagree - Strongly disagree     Please feel free to provide us with feedback on why you chose this answer.  [free text] |
